# Structural motifs recurring in different folds recognize the same ligand fragments

**DOI:** 10.1186/1471-2105-10-182

**Published:** 2009-06-15

**Authors:** Gabriele Ausiello, Pier Federico Gherardini, Elena Gatti, Ottaviano Incani, Manuela Helmer-Citterich

**Affiliations:** 1 Centre for Molecular Bioinformatics, Department of Biology, University of Rome "Tor Vergata", Via della Ricerca Scientifica, 00133 Rome, Italy; 2 C4T – Colosseum Combinatorial Chemistry Centre for Technology c.o. Department of Chemistry, University of Rome "Tor Vergata" Via della Ricerca Scientifica, 00133 Rome, Italy

## Abstract

**Background:**

The structural analysis of protein ligand binding sites can provide information relevant for assigning functions to unknown proteins, to guide the drug discovery process and to infer relations among distant protein folds. Previous approaches to the comparative analysis of binding pockets have usually been focused either on the ligand or the protein component. Even though several useful observations have been made with these approaches they both have limitations. In the former case the analysis is restricted to binding pockets interacting with similar ligands, while in the latter it is difficult to systematically check whether the observed structural similarities have a functional significance.

**Results:**

Here we propose a novel methodology that takes into account the structure of both the binding pocket and the ligand. We first look for local similarities in a set of binding pockets and then check whether the bound ligands, even if completely different, share a common fragment that can account for the presence of the structural motif. Thanks to this method we can identify structural motifs whose functional significance is explained by the presence of shared features in the interacting ligands.

**Conclusion:**

The application of this method to a large dataset of binding pockets allows the identification of recurring protein motifs that bind specific ligand fragments, even in the context of molecules with a different overall structure. In addition some of these motifs are present in a high number of evolutionarily unrelated proteins.

## Background

The understanding of the determinants of molecular recognition between proteins and small molecules is still an elusive goal in structural bioinformatics. Different proteins bind their cognate ligands with different degrees of specificity and such differences have important functional implications in a multitude of biological processes such as drug metabolism[1], the immune response[2] and many more. Understanding the structural grounds of these differences is therefore of paramount importance for both the precise and complete functional annotation of proteins and for the development of selective and active binders.

The increasing number of protein structures of unknown function determined in structural genomics projects[3] has prompted the development of a number of methods aimed at annotating ligand binding sites using structural information only [4-9]. Even when proteins belong to well characterised protein families, their ligand binding specificity is not trivial to identify and cannot simply be transferred by homology[10].

In order to understand the rules underpinning the interaction of proteins with small ligands, a wealth of information can be derived from the comparative analysis of binding pockets of known structure. Such analysis can be performed starting from either the ligand or the protein. In the former case a number of protein pockets that bind a ligand of interest are selected. The ligand moieties are subsequently superimposed in order to identify similarities and differences in the neighbouring protein atoms. This approach reveals some interesting relations [11-16], but necessarily limits the analysis to pockets that bind ligands with an overall similar structure, since these are used as a reference to guide the superimposition of the binding pockets.

Conversely, if the analysis starts from the protein side, two approaches can be conceived. The first possibility is to compare a family of homologous proteins with diverse binding specificities with the aim of correlating binding site variations with the presence or absence of specific chemical groups in the context of similar ligand molecules. Indeed detailed structural knowledge of a binding site is routinely used to engineer mutants with altered specificity [17-22]. Najmanovich et al.[23] investigated the extent to which binding site similarities correlate with small-molecule binding profiles in the sulfotransferase family. They concluded that proteins with similar binding profiles show similarity in their binding site. Conversely, given the current state of knowledge about molecular recognition, it is difficult to use binding site similarity to infer the specificity of a protein.

The alternative approach is to mine the Protein Data Bank, looking for binding motifs which are present in evolutionarily unrelated proteins. Since such motifs have evolved independently multiple times, they should represent favourable modes of interaction between protein residues and ligand moieties [24-27]. Indeed a number of sequence-independent local structural comparison methods have been applied to the analysis of protein binding sites [28-30]. The results of these experiments showed that the similarity of portions of two binding sites can be ascribed to common chemical moieties in the ligand. However none of these analyses did systematically take the structure of the bound ligands into account during the comparison and such observations were made a posteriori by manual inspection. This is indeed the main shortcoming of all the "protein-centric" approaches: in order to assess the functional significance of a structural similarity, the identified motifs have to be analysed to check whether they establish analogous interactions with the chemical groups of the ligand.

Here we propose a novel methodology that combines the advantages of both approaches, without being impaired by the above-mentioned limitations. More specifically, we firstly compare the structures of a set of binding pockets to identify regions of local similarity. When one such region is found, we investigate whether the corresponding residues also interact with similar chemical groups in the ligand moiety.

To demonstrate the validity of our approach we performed a novel analysis of all the binding pockets belonging to the structures classified in the Structural Classification of Proteins (SCOP) database[31]. We focused our attention on structural motifs shared by proteins belonging to different folds. Indeed our approach, which is based on local as opposed to global similarities, is expected to be well tailored to investigate such cases. More than 600 structural motifs were identified, each one associated to a specific ligand fragment. Some of these motifs occur in more than 60 folds.

## Results and Discussion

### Description of the approach

Here we propose a new method to identify structural motifs in protein binding pockets in a ligand-dependent manner. Our approach does not require the proteins, or their bound ligands, to be similar. Moreover one does not have to make any a priori assumption about the existence of common structures in the binding pockets or the ligands. The procedure comprises two steps. We first identify local structural similarities shared by a set of input structures. Following this first step we have a number of binding pockets superimposed in space according to the presence of a shared structural motif. Subsequently we analyse the coordinates of the bound ligands looking for the largest common fragment that has a similar position in space.

Therefore, by taking into account the structure of both the binding pocket and the ligand, we first identify structural motifs whose functional significance is readily explained by the presence of the interacting ligand fragment. This approach is akin to decomposing a binding pocket in small patches each one interacting with a given chemical group of the ligand.

To demonstrate the usefulness of our approach we performed a comparative analysis of all the binding pockets in the PDB structures classified in SCOP. We focused especially on the cases which are difficult to analyse with traditional approaches, namely binding sites on proteins with different SCOP folds involved in binding similar as well as different ligands. It should be emphasised that such cases effectively represent the largest class when one considers all the possible pairs of binding pockets of known structure and have been largely neglected in previous works for lack of an appropriate comparison methodology.

Our method exploits the PDB data to its maximum because it enables the pair-wise comparison of structures containing different ligands that interact with different protein folds. In either case what we are looking for are small structural motifs (protein residues + interacting ligand fragments) which are used in multiple and possibly different contexts.

### Identification of similarities in binding pockets extracted from the PDB

We applied the method to the analysis of the 24402 PDB structures classified in the SCOP database. For each ligand we defined a binding pocket made up of all the neighbouring protein residues (see Methods). The above procedures identified 65467 binding pockets, mapping to 4050 different ligands.

The Query3d structural comparison algorithm was used to identify local similarities in the dataset of protein binding pockets. Since we were only interested in similarities between non-homologous structures, we did not compare with each other binding pockets belonging to proteins assigned to the same sequence cluster (at the 30% sequence identity level, using the sequence clusters available from the PDB website).

Following this procedure 2.5 × 10^9 ^comparisons were performed which resulted in the identification of 1.1 × 10^6 ^structural matches. To further guarantee that only similarities between non-homologous structures were included in our analysis we also discarded all matches between proteins belonging to the same CATH Architecture and those belonging to the same SCOP fold. These steps removed respectively 468315 and 298963 matches, bringing the total number to 362557. This set of matches involves only non-homologous proteins but is still redundant since the same pair of SCOP folds can appear multiple times in the results. In order to generate a non-redundant set we grouped together all the matches between the same pair of SCOP folds and involving the same pair of ligands (as defined by their 3-letter code). This last step produced a list of 8490 non-redundant structural similarities between non-homologous binding pockets.

**Figure 1:**
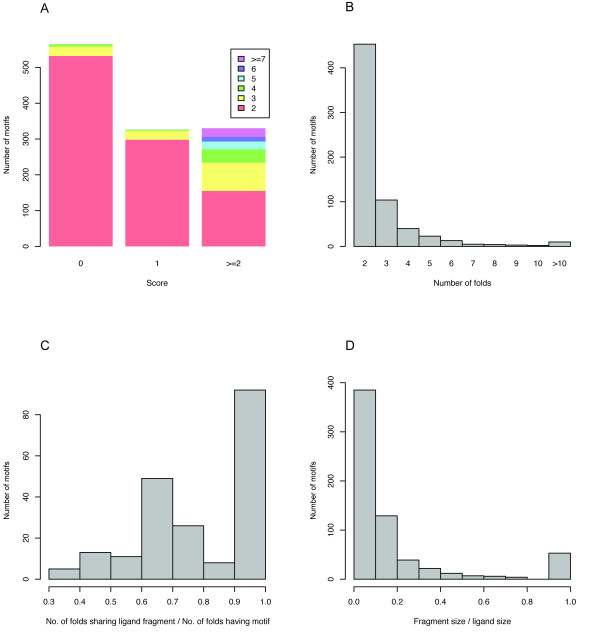
**Overview of the structural motifs identified in the PDB**. a) Distribution of the score associated to the presence of shared ligand fragments (see "Identification of shared ligand atoms") for the 1227 structural motifs identified. 570 motifs had a score of zero (i.e. they were not associated to a common ligand fragment) and were therefore discarded in the subsequent analyses. The bars are coloured according to the number of folds possessing the motif. b) Distribution of the number of folds sharing the motif for the 657 motifs with score > = 1 (i.e. associated to a definite ligand fragment). c) Distribution of the ratio of the number of folds having a structural motif associated to a shared ligand fragment to the total number of folds having the motif, i.e. including those for which the motif binds a different ligand fragment. This figure does not include motifs shared by two folds only since in that case the ratio is 1 by definition. d) Distribution of the ratio of the size of the common fragment to the size of the whole representative ligand for each motif. Values of 1.0 are associated to metal atoms.

After the identification of pair-wise similarities the FunClust algorithm was used to search for sets of matches involving residues common to different structures. This last procedure reduced the list of 8490 motifs present in two different folds to a non-redundant list of 1227 unique motifs located in a number of different folds [see Additional file 1]. More specifically, 104 motifs were found to map to three folds and 90 to 4–10 folds; a few exceptional cases involve from 17 up to 63 different folds (see Figure 1b) [see Additional file 2].

### Identification of shared ligand fragments

Having identified a set of structural motifs shared by two or more SCOP folds we analysed the coordinates of the bound ligands looking for the largest common fragment (subset of connected atoms) that has a similar position in space. To this end we devised a computationally efficient procedure to identify the common atoms in a set of superimposed ligands.

The ligands belonging to each binding pocket are first superimposed according to the 3D transformation (translation + rotation) used for the protein residues (see above). The algorithm then searches all the possible combinations of fragments (subset of connected atoms) using a recursive depth-first procedure and identifies the one with the highest score. The score is defined (see Methods) as a trade-off between the size of the fragment and the fact that it should be present in the highest possible number of bound ligands.

For the purpose of this application we were interested more in finding common motifs in proteins of completely different structure than in studying binding site variations in proteins of the same fold. Accordingly during the common-fragment identification step we selected only one representative structure for each fold possessing a given motif. This was done by identifying the common ligand fragments in all the possible combination of proteins possessing the motif (one protein per fold) and then selecting the combination that gave the fragment with the best score.

It should be noted that this approach of eliminating redundancy a posteriori guarantees to have the best result for the specific problem at hand. The opposite strategy, i.e. choosing one representative per fold at the beginning of the analysis, would have clearly been inferior since this selection would necessarily had to be done on arbitrary grounds (e.g. choosing the best resolution structure, or the one with the biggest ligand etc.).

Following this procedure 657 of the 1227 identified structural motifs were found associated to specific ligand fragments (score higher than 0), despite a high variability in the structure of the ligand as a whole. In addition to that a lesser number (570, i.e. 1227 – 657) of motifs were identified on the structure but no common fragment was found in the bound molecules (see Figure 1a). This last set of motifs was not considered any further. In 86% of the cases the common ligand fragment is associated to more than 60% of the folds possessing the motif, i.e. few binding pockets that have the motif do not share the common ligand fragment (see Figure 1c). Also in 78% of the cases such fragments are quite small, comprising less than 20% of the ligand atoms (see Figure 1d). Overall these figures suggest that the presence of specific residues in a binding pocket can be related to the identity and position of a number of ligand atoms. To further test this hypothesis we devised the following benchmark.

### Benchmark of the method

We devised a benchmark to test the assumption that underlies our method, i.e. that the presence of specific protein residues implies a discernible preference for certain ligand fragments. We identified a set of non-redundant pairwise structural similarities between binding pockets belonging to proteins of different folds (see Methods for a definition of the dataset). Our aim was to test whether the correspondences we identified on the ligand have a functional significance i.e. whether the ligand fragments that match are effectively those that are close to the residues comprising the structural motif.

Each protein structural similarity implies a 3D transformation (rotation + translation) of the binding sites and, accordingly, of the bound ligands. Using the LIGANDSCOUT[32] software we identified a total of 3161 pharmacophoric groups (steric or electronic features possessed by a ligand molecule necessary to ensure its interactions with the binding pocket) in the 210 ligands included in our Benchmark dataset. The pharmacophoric features were distributed as follows: 1998 H-bond acceptors (A), 299 H-bond donors (D), 482 H-bond donors or acceptors (DA), 348 negative ionizable areas (N), 34 positive ionizable areas (P). We did not take into account aromatic and hydrophobic features because they could not be effectively captured by our local comparison methods. Indeed such features are primarily due to the size and shape of the binding pocket and its electrostatic nature (polar/non-polar). These characteristics of a binding pocket cannot be described by local comparison methods that, by their nature, focus on the 3D position of a small set of residues or chemical groups. We think that such motifs are more suited to the description of hydrogen-bond patterns between the ligand molecule and the protein residues. Accordingly we limited our analysis to these pharmacophoric features.

**Figure 2:**
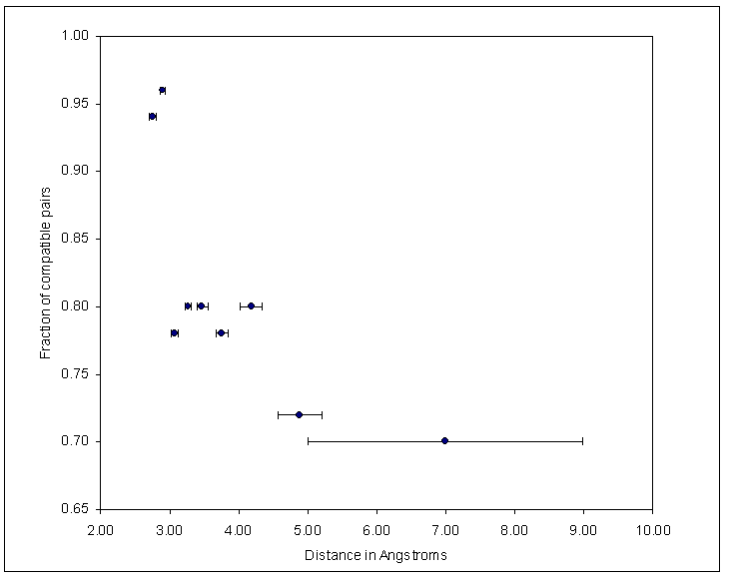
**Relationship between the correspondence of ligand pharmacophores and their distance from the structural motif on the protein**. The list of matching pharmacophores was sorted according to their distance from the centroid of the structural motif. This sorted list was then divided in nine bins of 50 pharmacophores each. The value on the x axis represents the average distance in each bin (the bars represent the standard error). The value on the y axis is the ratio of the number of pharmacophore pairs with comparable chemical roles to the total number of pharmacophore pairs in each bin.

If the correspondences we identify have a functional significance, one would expect the features that match after applying the above-mentioned 3D transformation to be located close to the residues identified in the structural comparison. In other words, the matching pharmacophores must be the ones that interact with the residues involved in the superposition as opposed to another pair of pharmacophoric points, which has no relationship to them. We counted the number of points having at least one other pharmacophore in the superimposed ligand at a distance of less than 0.8 Angstroms. We identified 450 pairs (out of 3161) of pharmacophoric points matching this criterion. To correct for the fact that a higher number of ligand atoms is expected as one moves closer to the protein surface we measured the ratio of the number of matching pharmacophores which give similar interactions with the protein atoms to the total number of matching pharmacophores. We found 364 compatible and 86 non-compatible pairs. We then sorted these couplets of pharmacophores according to their distance from the geometric centroid of the residues comprising the structural match. This sorted list was divided in 9 bins, each containing 50 pairs. The plot in figure 2 shows the fraction of compatible pairs of pharmacophores in each bin as a function of their average distance from the residues identified in the structural match. There is a clear trend for similar pharmacophores in the ligands to be located close to the structural motif. Moreover the proportion of compatible pairs reaches a plateau when the distance exceeds 5 Angstroms. This shows that the correspondences we identify are functionally significant, because the shared ligand atoms are those that effectively interact with the residues involved in the structural similarity.

### Manual classification of the identified motifs

**Figure 3:**
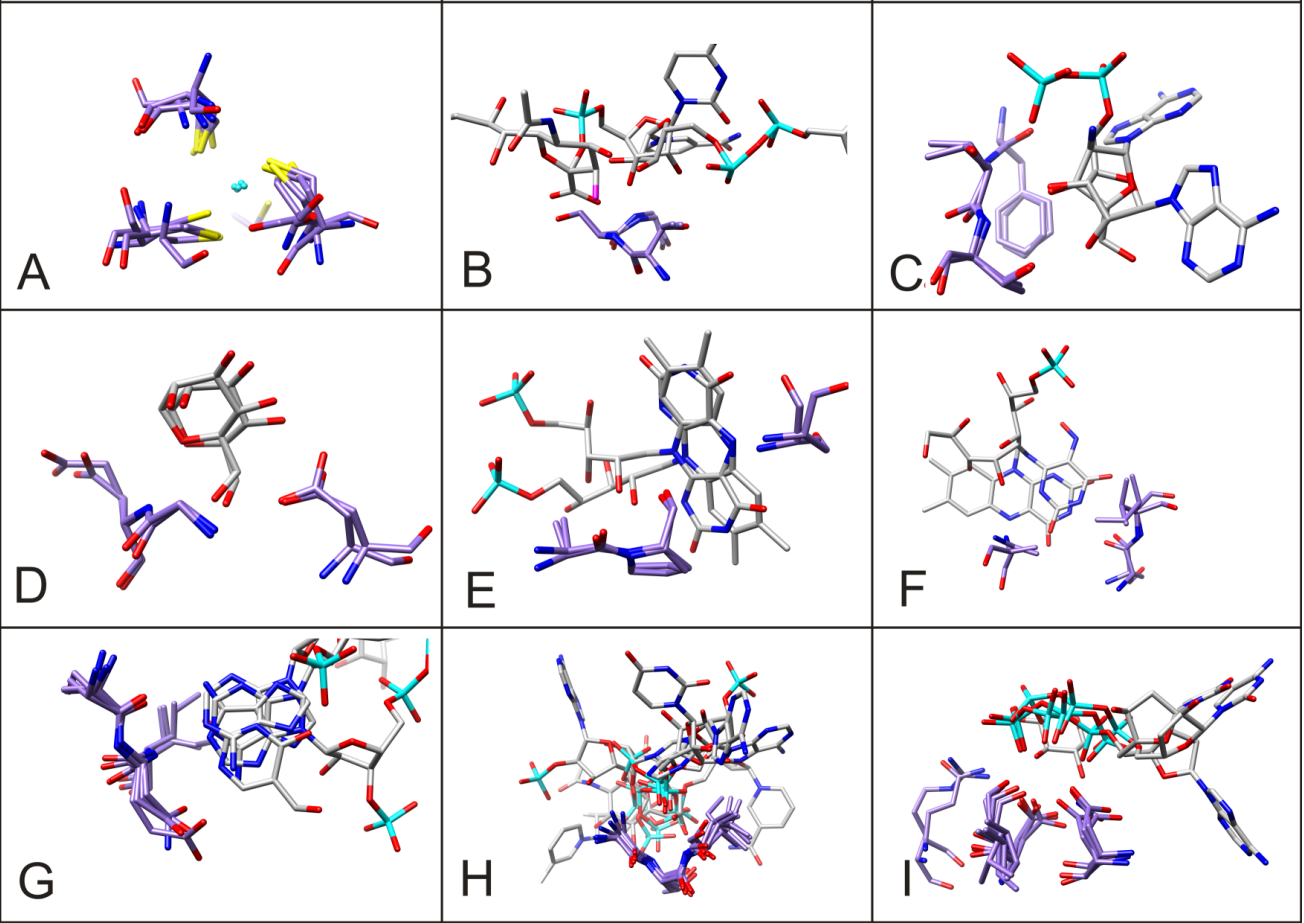
**Examples of identified structural motifs**. Nine different structural motifs with their ligands are shown. Only the residues comprising the motif and their ligands are shown in the picture. All the atoms are coloured by type. Carbon atoms of the ligands are in white and carbon atoms of the protein are in light purple. (A) Zinc binding motif (id 112) involving six folds all using three cysteine residues to bind a ZN atom. (B) Ribose binding element (id 11) using a T-[AS]-G motif to interact with the O4 of ribose in two nucleotide-like ligands. (C) Ribose binding motif (id 472) using a phenylalanine and two threonine residues to interact with ribose in two different nucleotides. The two ribose fragments are coplanar but lay in opposite orientations. (D) Mannose binding motif (id 378) comprising two collinear residues G-[DE] and a third aspartic acid residue in position +4 in a fold and -134 in the other. (E) A Flavin binding motif (id 426). Residues A-P-X-[AS] interact mainly with the central ring of the two flavin mononucleotides. The two molecules are coplanar but have different orientations. (F) A motif interacting with pyrimidine ring derivatives (id 429); residues A-X(n)-[SA]-[IV] interact in one case with the flavin and in the other with a flavin precursor. (G) Nucleobase binding motif (id 634). Four different folds share the three residue motif [AS]-[DEN]-[IV] that makes hydrogen bond contacts with the pyrimidine ring of four different purines. (H) Phosphate binding motif (id 31) present in six different folds possessing the G-[STA]-[IVL] pattern. The interacting fragment is a phosphate group coming from a variety of ligands. (I) Phosphate binding motif (id 398): two aspartic acids and a leucine or isoleucine residues interact with the oxygen atoms of different phosphate groups in three different folds. In a fourth fold a slightly different motif interacts with an oxygen atom of a β-D-xylopyranose molecule.

The 330 motifs with a score greater than 1 have been manually analysed in order to categorise the types of ligand fragments recognised. The results of this classification show that the vast majority of the analysed structural motifs are involved in the binding of anions (phosphate and carboxyl groups) and nucleotides, 215 and 35 motifs respectively. Other highly represented motifs bind metals, 14, and heme groups, 10. Overall these figures confirm that our methodology is sound. Most of our results comprise motifs that are already known in the literature as having widespread occurrence in fold space (i.e. metal binding sites[33] and phosphate binding sites[24]). 28 of the identified motifs are listed that recognize important and widespread biological ligands [see Additional file 3]. In particular, hexoses are associated to 4 motifs, flavin to 4 motifs, nucleobases to 10 motifs, riboses to 6 motifs and heme to 4 motifs. In figure 3 a number of significant motifs are described. The motifs have been chosen in order to represent the variability of recognized ligand fragments. For the sake of clarity in the graphical representation small motifs and motifs shared by only two fold have been preferred.

**Figure 4:**
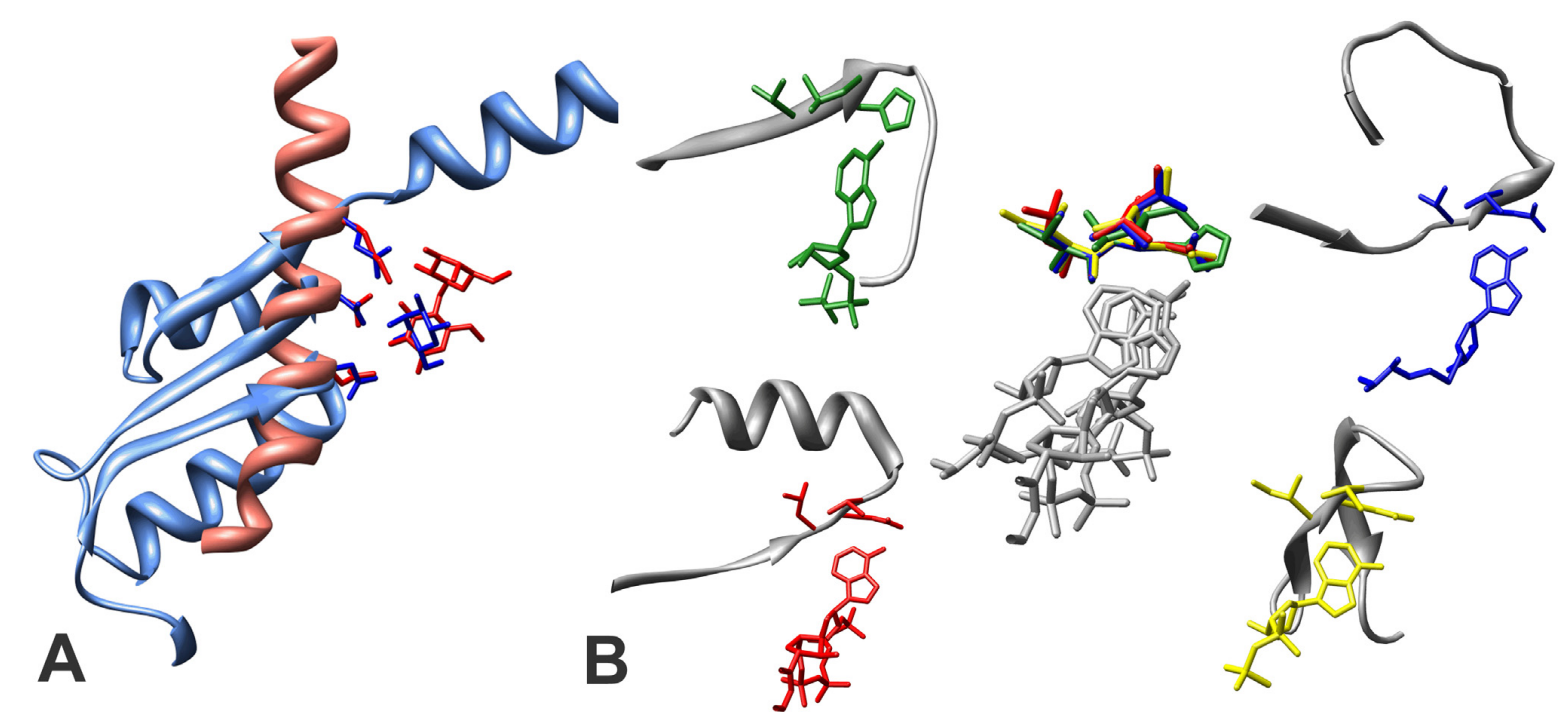
**Examples of two identified structural motifs**. (a) A three-residue structural motif used to bind a sugar in a D-allose binding protein (PDB code: 1rpj in light blue) and in a Glycolipd Transfer Protein (PDB code: 1sx6 in salmon). Only part of the protein chains is displayed. Residues Asn 52 and Asp 48 (in red) of 1sx6, similarly to the matching residues Gln 247 and Asp 227 (in blue) of 1rpj, are hydrogen bonded to the hydroxyl groups of the sugar ligands (in red and blue). (b) A three-residue nucleotide binding motif found in four proteins: a methyltransferase from Dengue virus (1l9k in blue: val130, asp131 and val132), saccharopine reductase from Magnaporthe grisea (1e5q in red: leu54, asp55 and val56), riboflavin kinase from Schizosaccharomyces pombe (1n07 in green: val97, his98 and leu99) and a Group II Chaperonin from Thermococcus (1q3s in yellow: ile479, asp480 and val481). In the corners of the figure each structure is represented alone in grey with the residues comprising the structural motif and the ligand in colour. In the centre all the four structures are shown superimposed on their structural motif. The residues in the motif are coloured while the four ligands are in grey.

Several structural motifs resulting from our analysis were, to the best of our knowledge, previously unknown. We describe here two specific examples. Figure 4a displays a structural motif used to bind a sugar by an E. Coli D-allose binding protein (PDB code: 1rpj). This motif is also present in a human Glycolipid Transfer Protein (PDB code: 1sx6). The figure clearly shows that these two proteins have completely different folds, indeed they also belong to different SCOP classes (α/β and all α respectively). In 1rpj, the ligand binding cleft is located in a hinge region between two domains, composed of a three stranded beta-sheet. Asp227 and Gln247, together with ten other residues, form a network of hydrogen bonds with the hydroxyl groups and ring oxygen atoms of the sugar[34]. The structure of 1sx6 is completely different: in this case the binding site is sandwiched between two layers of alpha helices[35]. Asn52 and Asp48, similarly to the matching residues Gln247 and Asp227 of 1rpj, are hydrogen bonded to the hydroxyl groups of the sugar.

The second example is shown in Figure 4b and shows a nucleotide binding motif found in four proteins: a methyltransferase from Dengue virus (1l9k), saccharopine reductase from Magnaporthe grisea (1e5q), riboflavin kinase from Schizosaccharomyces pombe (1n07) and a Group II Chaperonin from Thermococcus (1q3s). These structures all belong to different folds and indeed, as the figure clearly shows, they are completely different despite a remarkable similarity of the binding motifs. In each case there is a conserved interaction between the backbone of a hydrophobic residue (valine in 1q3s, 1e5q, 1l9k and leucine in 1n07) and the N1 atom of the nitrogen base. The same leucine of 1n07 also interacts with the N6 atom while this role is fulfilled by an aspartate residue in 1e5q and by a water molecule in 1l9k.

These two examples show the validity of our approach. By looking at similarities of portions of the binding pockets we were able to identify structural motifs that recognise specific chemical groups in different contexts.

## Conclusion

This work details a novel methodology to study small molecules recognition by protein structures. The main point of our method is that the structures of the binding pocket and the ligand are both considered when comparing a set of proteins. Following this approach we identify structural motifs whose functional significance is readily defined by the presence of common fragments in the ligand moiety.

An all-against-all comparison of all the binding pockets in the PDB structures classified in SCOP resulted in the identification of more than 600 structural motifs, each one associated with a definite ligand fragment and identified in at least two different protein folds. The validity of our method is proved by the fact that we are able to identify known structural motifs together with a number of novel ones. Some of these motifs have an exceptionally widespread occurrence, being present in more than 60 protein folds. In particular we found that the motifs of more widespread occurrence are those involved in binding anions (phosphate and carboxylate), metals and nucleotides.

In analogy to short linear motifs[36], short structural motifs are also subject to severe overprediction, because of the lack of an adequate statistics[4] and cannot be efficiently used in a classic 3D pattern matching approach. The advantage of our method lies in the fact that the motifs we identify are associated to specific ligand fragments and therefore their functional significance is readily apparent. Once a motif has been identified and validated in this way it can be used to screen a target structure, irrespectively of whether the protein has been crystallized with a ligand or not. In other words the ligand is only needed for defining the motif, which can then be used by itself.

One important outcome of this work is the high-throughput identification of the functional 3D motifs in different protein folds. These data offer the opportunity to discuss the evolutionary origin and history of the 3D motifs. One possibility is that this distribution arose by convergent evolution; in this case, we must stress the somehow unexpected plasticity displayed by the evolutionary unrelated protein folds able to host the 3D motifs. An opposite scenario is also possible, the one with short structured peptides, in relatively limited number, that gave rise to different proteins in a diverging process[37]. If so, the analysis of the motifs distribution could be used to trace the evolutionary neighbourhood of protein folds now considered unrelated.

Moreover, the focus on local features of the protein and ligand can be used to study variations in binding specificity in groups of homologous proteins as such changes are likely guided by specific residue substitutions in the binding site.

## Methods

### Binding pockets dataset

We used all the PDB structures classified in SCOP. The mapping from single residues to SCOP domains was obtained from the MSD[38] database and all the following numbers refer to such set of PDB structures. A ligand binding pocket database was constructed starting from the mmCIF[39] coordinate files as follows. We selected in the mmCIF file all the non-protein entities excluding DNA fragments and a set of small molecule ligands usually present in crystallization buffers. Multiple identical ligands in the same structure file were distinguished by virtue of their mmCIF asym code. For each ligand we defined a binding pocket made up of all the protein residues that had an atom whose distance from any atom of the ligand was less than 3.5 Angstrom.

### Structural comparison of the binding pockets

In the first step of our procedure we used the Query3d structural comparison algorithm to compare the binding pockets. Query3d identifies the largest set of residues in two protein structures that possess a good biochemical similarity (as defined by a substitution matrix) and can be superimposed under a given RMSD threshold. The matching process is completely sequence independent. The RMSD is calculated using a two-point representation of each residue, consisting of the C-alpha and the geometric centroid of the side-chain geometric centre. The RMSD threshold was set to 0.4, 0.8, 1.3 and 1.5 Angstrom for matches comprising 3, 4, 5 and 6 or more residues respectively; only residues with a similarity score of at least 1 in a BLOSUM62 matrix were allowed to match. In order to find the matches, an exhaustive depth-first search is performed exploring all the possible combinations of neighbour aminoacids belonging to the two different proteins. Two residues are considered neighbours if the distance between their C alpha atoms is less than 7.5 Angstroms.

The choice of RMSD threshold was based on several works already performed in our group using the Query3d algorithm (see e.g. [40-43]), with an improvement suggested by the requirement of adjusting the RMSD threshold according to the number of aligned residues. Indeed the 0.7 Angstrom limit that was used in several works [40-42] was found to produce many non-significant matches when the similarity involves only three residues; on the other hand, many known examples of convergent evolution can be identified only with higher RMSD thresholds (up to 1.5 Å [43]). Accordingly for the present work we used a variable threshold spanning the range 0.4–1.5 Angstrom. The minimum size of a structural match is three residues which is a very low threshold and therefore unlikely to miss significant similarities.

Subsequently we used the FunClust algorithm[44] to search for sets of matches involving residues common to different structures. To this end, FunClust builds a graph in which every node represents a match between a pair of structures. Edges are drawn between nodes representing the same match (same residues) of a structure with two different ones. This graph is analyzed by a fast and simple procedure that searches for the highest scoring set of connected nodes in the graph containing no more than one match between the same pair of structures. The score is given by the number of residues in common between all the matches multiplied by the number of matches belonging to the cluster. Given a set of structures FunClust guarantees to recover the largest local structural motif shared by the highest possible number of these structures.

### Identification of shared ligand fragments

The common fragment identification procedure is applied to the ligands which have been superimposed according to the same 3D transformation (rotation + translation) that was used for the structural motif. The procedure is complicated by the fact that each motif in a fold corresponds to a set of different ligands and not just to a single one. This is because a motif maps to proteins belonging to different folds, as well as to multiple proteins of the same fold, bound to different ligands.

To identify the best fragment each ligand is compared pairwise with all the other ligands that are associated with a different fold. For each comparison the largest common fragment is identified. All the identified fragments are then joined together. The details of the matching and merging procedures are described below. For each structural motif these procedures are applied to all the ligands, one at a time. The algorithm then returns the fragment with the highest score.

For the purpose of pairwise matching the ligands are described as graphs. Each heavy atom is a node and edges connect atoms which are connected in the molecular structure of the ligand. Each pair of nodes constitutes a "seed match" that is extended using a recursive depth-first procedure. During each step a new pair of atoms is added to the match choosing among all the atoms connected to those that already belong to the match. The recursion goes back one level each time a pair of non-identical atom types is chosen, or the distance between the pair of atoms is higher than 1.0 Angstrom or the global RMSD of the match exceeds a 1.5 Angstrom threshold. C and P are considered as a single atom type and therefore they can be paired. This was done in order to match -PO4 and -COO groups since they are often bound by similar structural motifs[45]. At the end of the procedure the longest identified common fragment (match) is returned. All the identified fragments are then mapped on the ligand under analysis and merged in a single fragment. The common fragment is defined as the union of all the atoms in the ligand that are present in at least one fragment. The score of the fragment is given by the sum of the squared scores of its constituent atoms.

The score of each atom is the number of different fragments in which the atom was present (counting no more than one fragment for each different fold). C and P atoms do not contribute to the score. Therefore the maximum possible score for a single atom is equal to number of folds sharing the structural motif minus one. In computing the total score of the fragment we took the square of the single atom score in order to weigh the number of different folds in which the atom was found more than the size of the fragment.

We did not set any threshold for the minimum number of superimposed ligand atoms. However for practical reasons the manual analysis of motifs was limited to the 330 motifs with a score greater than one. In practice this means that motifs involving three folds were manually inspected when they had at least one common atom on the ligand. Conversely, for motifs present in only two folds, we required at least two atoms of the ligands to be superimposed.

### Benchmark dataset

In order to benchmark the method, we used Query3d to obtain a set of pairwise similarities between all the proteins in the dataset. We also applied the same procedure, described above, in order to remove matches between the same SCOP folds and eliminate redundancy. However we imposed a number of additional requirements.

First we selected only structural matches comprising 3 residues because larger similarities may imply globally similar orientations of the ligand which would generate correspondences between portions of the molecules irrespective of the structural motif and therefore alter our statistics. For the same reason, we selected only similarities associated to ligands of different structure (i.e. sharing a Tanimoto score lower than 0.85, calculated using SuperLigands[46]). These precautions guarantee that the observed similarities between fragments of the ligands are due neither to an overall similar structure and orientation of the whole ligand molecule nor to the fact that the proteins involved have the same fold.

We also selected only the binding pockets whose ligands have at least 4 superimposed atoms (i.e. less than 1 Angstrom distance), to have a high number of superimposed ligand atoms. The above criteria reduce the set of structural matches that can be used; accordingly we adopted a more lax threshold of 1 Angstrom RMSD for the structural comparison in order to have a sample of adequate size.

## Authors' contributions

GA conceived the study and carried out the fragment comparison, PFG conceived the study, drafted the manuscript and carried out the clustering, EG carried out the structural comparison and classified the motifs, OI participated in the analysis of the ligands, MHC participated in the design of the study and in its coordination. All authors read and approved the final manuscript.

## Supplementary Material

Additional file 1 **Complete list of all the identified structural motifs with score > 1**. For each motif a representative PDB structure per row is shown for each different SCOP fold in which the motif is present. 1) The id number of the motif. 2) Manual classification of the matching fragment: a = planar ring (also in nucleotides), e = heme, h = possibly distant homologues (discarded), m_xx = metal (where xx represents the metal id), n = nucleotide, o = oxygen atom, p = phosphate and/or carboxyl group, r = ribose, s = not classifiable, z = sugar, *) not manually classified (score = 1). 3) SCOP fold id of the representative structure. 4) The PDB id of a representative protein structure having the structural motif. 5) The PDB chain id of the residues belonging to the motif. 6) The id of the motif residues. 7) The PDB id of the ligand that is present in the binding pocket of the representative structure. 8) The ids of the ligand atoms that are shared among the representative structures. For the leading structure (the first listed for each motif), all atoms in common with all the other structures of the motif are reported. For all the other structures, only the atoms shared with the leading structure are shown. Click here for file

Additional file 2 **Score and fold distribution of the identified structural motifs**. 1) The id number of the motif. 2) Manual classification of the matching fragment: a = planar ring (also in nucleotides), e = heme, h = possibly distant homologues (discarded), m_xx = metal (where xx represents the metal id), n = nucleotide, o = oxygen atom, p = phosphate and/or carboxyl group, r = ribose, s = not classifiable, z = sugar, *) not manually classified (score = 1). 3) Number of different SCOP folds having at least one structure with the motif. 4) Number of different SCOP folds having at least one structure with the motif and a common fragment on their ligands. 5) Score of the motif. Click here for file

Additional file 3 **List of selected local structural motifs identified by the method grouped by the type of recognized ligand**. 1) The identificative number of the motif. If more than a number is reported, this means the structural motifs resulted to be the same after visual inspection. 2) The name of the SCOP folds having at least one structure containing the motif. 3) The PDB id of a representative protein structure belonging to the fold and sharing the structural motif. 4) The PDB chain id of the residues belonging to the motif. 5) The id of the motif residues. 6) The PDB id of the ligand present in the binding pocket of the representative structure. 7) The ids of the ligand atoms that are shared among the representative structures. For the leading structure (the first listed for each motif), all atoms in common with all the other structures of the motif are reported. For all the other structures, only the atoms shared with the leading structure are shown. Click here for file
