# Supplementary material for: Structural motifs recurring in different folds recognize the same ligand fragments
Source: BMC Bioinformatics. 2009 Jun 15;10:182. doi: 10.1186/1471-2105-10-182 (PMC2704211; doi:10.1186/1471-2105-10-182)
Supplement: Additional file 3 — List of selected local structural motifs identified by the method grouped by the type of recognized ligand. 1) The identificative number of the motif. If more than a number is reported, this means the structural motifs resulted to be the same after visual inspection. 2) The name of the SCOP folds having at least one structure containing the motif. 3) The PDB id of a representative protein structure belonging to the fold and sharing the structural motif. 4) The PDB chain id of the residues belonging to the motif. 5) The id of the motif residues. 6) The PDB id of the ligand present in the binding pocket of the representative structure. 7) The ids of the ligand atoms that are shared among the representative structures. For the leading structure (the first listed for each motif), all atoms in common with all the other structures of the motif are reported. For all the other structures, only the atoms shared with the leading structure are shown. [file 1471-2105-10-182-S3.doc]

| **Motif ID (1)** | **SCOP Fold (2)** | **Representative PDB Structure (3)** | **Chain ID (4)** | **Binding Residues (5)** | **Ligand ID (6)** | **Ligand fragment atoms (7)** |
| --- | --- | --- | --- | --- | --- | --- |
| **Hexose** | | | | | | |
| 30* | Periplasmic binding protein-like I | 1rpj | A | N201 D227 Q247 | ALL | C2* C3* C4* C5* O3* O4* O5* |
|  | Glycolipid transfer protein, GLTP | 1sx6 | A | D48 N52 K55 | LAT | C3' C2' C1' O2' C4' O5' |
| 378 | beta-Prism I | 1vbp | B | D141 G137 D138 | MAN | C1 C2 C3 C4 C5 C6 O2 O3 O4 O5 O6 |
|  | Concanavalin A-like lectins/glucanases | 1q8q | A | D86 G220 E221 | MAN | C4 C5 O5 C1 C2 O2 C6 O6 C3 O4 O3 |
| 507 | beta-Prism II | 1kj1 | A | Q26 D28 N30 | MAN | C1 C2 O1 O2 |
|  | C-type lectin-like | 1kx1 | F | K182 E185 N205 | MAN | C2 O2 C3 O3 |
| 1099 | Thymidylatesynthase/dCMP hydroxymethylase | 1hvy | D | Q214 S216 G217 | UMP | N3 C4 O4 |
|  | UDP-Glycosyltransferase/glycogen phosphorylase | 1p4h | A | E672 S674 G675 | CR6 | C9 O9 N1 |
| **Flavin** | | | | | | |
| 419 | TIM beta/alpha-barrel | 1h50 | A | P24 L25 T26 | FMN | N3 C4A N5 C5A C6 C9A N10 C10 C1* C2* O2* C3* O3* |
|  | Reductase/isomerase/elongation factor common domain | 1ep2 | B | P54 I55 S56 | FAD | O3* C3* C2* O2* C1* N10 C9A C5A N5 C4A C4 C10 |
| 712 | NAD(P)-binding Rossmann-fold domains | 1equ | A | T140 G141 S142 | NAP | NO2 NO3* |
|  | FAD-binding domain | 1hsk | A | A152 G153 A154 | FAD | O2* |
| 429 | Split barrel-like | 1usf | A | A37 S53 I54 | FMN | C2 O2 N3 C4 O4 |
|  | Lumazine synthase | 1c2y | R | A56 A80 V81 | LMZ | C2 N3 O2 C4 O4 |
| 426 | TIM beta/alpha-barrel | 1vhn | A | A10 P11 A13 | FMN | C4 C4A N5 C5A C6 C9A N10 C10 C1* C2* O2* C3* O3* |
|  | Split barrel-like | 1eje | A | A44 P45 S47 | FMN | N10 C10 C4A C4 N5 C5A C9A C1* C6 C2* O2* C3* O3* |
| **Nucleobase** | | | | | | |
| 263* | S-adenosyl-L-methionine-dependent methyltransferases | 1l9k | A | V130 D131 V132 | SAH | C5* C4* O4* C3* O3* C2* O2* C1* N9 C8 N7 C5 C6 N6 N1 C2 N3 C4 |
|  | NAD(P)-binding Rossmann-fold domains | 1e5q | A | L54 D55 V56 | NDP | AC5* AC4* AO4* AC1* AC2* AC3* AN9 AC4 AN3 AC2 AN1 AC6 AC5 AN6 AN7 AC8 AO3* AO2* |
|  | Reductase/isomerase/elongation factor common domain | 1n07 | B | V97 H98 L99 | ADP | C2* C1* N9 C4 C5 C6 N1 C8 C2 N7 N3 N6 |
|  | GroEL equatorial domain-like | 1q3s | H | I479 D480 V481 | ADP | C5 C6 N1 C2 N6 N3 C4 N7 N9 C8 |
| 634 | DHS-like NAD/FAD-binding domain | 1o96 | F | A305 D306 I307 | FAD | AO4* AC2* AO2* AC1* AN9 AC8 AN7 AC5 AC6 AN6 AN1 AC2 AN3 AC4 |
|  | Uracil-DNA glycosylase-like | 1oe6 | A | S145 E146 V147 | HMU | C2 N1 |
|  | ClpP/crotonase | 1ey3 | C | A98 D99 I100 | DAK | C2 N1 C6 N3 N6 |
| 225 | Ribosomal protein S5 domain 2-like | 1oj4 | B | G106 G107 N110 | ANP | PA O1A O2A O3A O5* C5* C4* O4* C3* O3* C2* O2* C1* N9 C5 C6 N6 C4 |
|  | Tubulin nucleotide-binding domain-like | 1rq7 | A | G18 G19 N22 | GDP | PA O1A O2A O3A O5* C5* C4* C3* C2* O3* O2* O4* C1* N9 C4 C5 C6 N1 |
| 682 | Ribosomal protein S5 domain 2-like | 1h74 | B | N62 V63 S101 S98 | SAP | O4* N9 C8 N7 C5 C6 N6 N1 C2 N3 C4 |
|  | NAD(P)-binding Rossmann-fold domains | 1rwb | A | D65 V66 T115 A93 | NAD | AC5 AC4 AN9 AC8 AN7 AN3 AC2 AN1 AC6 AN6 |
| 1076 | Ribokinase-like | 1gqy | A | H127 G128 N295 | ACP | N9 C8 N7 C5 C6 N6 N1 C2 N3 C4 |
|  | ATPase domain of HSP90 chaperone | 1th8 | A | H54 G55 D81 | ADP | C6 N6 C5 C4 N9 N3 C2 N1 C8 N7 |
| 1080 | Adenylylcyclase toxin (the edema factor) | 1k90 | B | H577 G578 T579 | 3AT | C1* N9 N7 C5 C2 N3 C4 |
|  | Isocitrate/Isopropylmalate dehydrogenase-like | 1iso | _ | H339 G340 T341 | NAD | AC2 AN3 AC4 AN9 AC1* AC5 AN7 |
| 1066 | Nucleotide-diphospho-sugar transferases | 1eyr | A | L10 A11 R12 | CDP | N1 C2 N3 C1* |
|  | Dihydrofolate reductase-like | 1ohk | _ | L75 S76 R77 | NDP | AN3 AC2 AC4 AN1 |
| 1137 | ATP-grasp | 1fvi | A | P26 K27 I28 | AMP | C1* N9 C8 N7 C5 C4 |
|  | Bacillus chorismate mutase-like | 1knj | A | P103 K104 M105 | C5P | N3 C4 C5 C2 N1 C6 |
| 1029 | PRTase-like | 1pzm | A | A177 F178 V179 | 5GP | N7 C5 N3 C4 |
|  | Phosphorylase/hydrolase-like | 1nc3 | B | A150 F151 I152 | FMC | N3 C4 C5 N7 |
| 39 | DHS-like NAD/FAD-binding domain | 1m2j | A | N211 P212 G185 G20 S187 G22 | APR | N1 C2 N3 C4 C5 C6 N7 C8 N9 C1* C2* O2* C3* O3* O4* C4* C5* O5* PA O1A O2A O3A PB O1B O2B RO5* RC5* RO4* RC3* RC4* |
|  | Formate dehydrogenase/DMSO reductase, domains 1-3 | 1fdi | _ | D202 P203 G174 G296 N176 G298 | MGD | O2A PA O3B PB O2B O5' C5' C4' C3' C2' C1' O4' N9 C8 N7 C5 C4 C6 N3 N1 C2 O2' O3' O3A O1A C10 C11 O1B C12 O11 |
| **Ribose** | | | | | | |
| 676 | NAD(P)-binding Rossmann-fold domains | 1ihx | B | G7 D32 P33 | SND | AP AO1 AO2 AO5* AC5* AC4* AO4* AC3* AO3* AC2* AO2* AC1* AN9 AC8 AN7 AC5 AC6 AN6 AN1 AC2 AN3 AC4 O3 NP NO1 NO2 |
|  | Formate dehydrogenase/DMSO reductase, domains 1-3 | 1fdi | _ | G174 D202 P203 | MGD | C4' O4' C1' N9 C4 N3 C5 C2' C6 C2 N7 O2' C8 N1 C3' C5' O3' O5' PB O2B O3B O1B PA O1A O3A |
| 277 | NAD(P)-binding Rossmann-fold domains | 1h6a | A | I157 L158 P159 | NDP | AO2 AO5* NC4* NO4* NC3* NO3* NC1* NN1 NC2 NC3 AOP1 |
|  | ALDH-like | 1o02 | B | I165 I166 P167 | NAD | AO3* AC3* AC4* AO4* AC1* AN9 AC4 AC5 |
| 11 | DHS-like NAD/FAD-binding domain | 1rlz | A | T131 A132 G133 | NAD | NO4* NC3* NC2* NO2* NC1* |
|  | Alpha-2,3/8-sialyltransferase CstII | 1ro7 | B | T131 S132 G133 | CSF | C2* C3* C4* O3* O4* |
| 472 | ATPase domain of HSP90 chaperone | 1thn | A | F97 T98 T99 | ADP | C5* C4* O4* C2* O2* C1* |
|  | ValRS/IleRS/LeuRS editing domain | 1obc | A | F246 T247 T248 | 2AD | C1* O4* C4* C2* C5* O5* |
| 630 | DHS-like NAD/FAD-binding domain | 1m2g | A | G185 N211 D213 | APR | N1 C2 N3 C4 C5 C6 N6 N7 C8 N9 C1* C2* O2* C3* O3* O4* C4* C5* O5* PA O1A O2A O3A PB RO5* |
|  | Activating enzymes of the ubiquitin-like proteins | 1r4n | D | G55 D79 D81 | ATP | C4 N9 C8 N7 C1* O4* C2* C3* O3* C4* O2* C5 C5* O5* C6 N6 N3 |
| 632 | S-adenosyl-L-methionine-dependent methyltransferases | 1q0t | B | D50 I51 Q52 | SAH | C4* O4* C3* O3* C2* C1* N9 C8 N7 C5 C2 N3 C4 |
|  | HIT-like | 1st4 | A | D205 L206 K207 | GTA | C4A O4A C1A N9 C4 N3 C3A C2A C8 C2 C5 O3A N7 |
|  | ClpP/crotonase | 1ey3 | B | D99 I100 K101 | DAK | N6 C6 N1 C2 C5 N7 |
| **Heme** | | | | | | |
| 127  290  96 | Multiheme cytochromes | 1qdb | B | C300 C303 H304 | HEM | All ligand atoms |
|  | Cytochrome c | 1mg2 | L | C57 C60 H61 | HEM | All ligand atoms |
|  | Common fold of diphtheria toxin… | 1e2z | B | C21 C24 H25 | HEC | All ligand atoms |
|  | Electron transport chains | 1dwl | B | C10 C13 H14 | HEC | All ligand atoms |
|  | Four-helical up-and-down bundle | 1gqa | D | C119 C122 H123 | HEC | All ligand atoms |
| 1220  65  1219  1223  1221  18 | Cytochrome c oxidase subunit I-like | 1fft | A | I417 H421 I424 | HEM | CHA CHB N A C1A C4A N B C1B N D C4D |
|  | Globin-like | 1gcw | C | L83 H87 L91 | HEM | CHC C4B N B C1B CHB C1C N C C4A N A |
|  | Heme-binding four-helical bundle | 1qlb | F | G133 H182 G183 | HEM | CHD C4C N C C1D N D C3C CAC |
|  | Chlorophyll a-b binding protein | 1rwt | E | L64 H68 W71 | CLA | CHA C1A C2A C4D N A C3A C4A CHB N D |
| 291 | Photosystem I subunits PsaA/PsaB | 1jb0 | B | R173 H176 H177 | CL1 | CHA CHD N A C1A N D C1D C2D C3D C4D |
|  | Multiheme cytochromes | 1h29 | C | K107 Y110 H111 | HEC | CHB C4A N A C1A C2A C3A CMA C1B N B |
